# Genotype classification and pathogenicity of infectious bursal disease virus circulating in vaccinated broiler chicken farms

**DOI:** 10.1007/s11259-024-10468-z

**Published:** 2024-07-30

**Authors:** Samah M. Mosad, Mona M. Elsayed, Enas M. Hammad, Basma M. Hendam, Hanaa S. Ali, Abdelfattah H. Eladl, Mohamed A. Saif

**Affiliations:** 1 Department of Virology, Faculty of Veterinary Medicine, Mansoura University, Mansoura, Egypt; 2 Department of Hygiene and Zoonoses, Faculty of Veterinary Medicine, Mansoura University, Mansoura, Egypt; 3 Department of Poultry Diseases, Agricultural Research Center, Animal Health Research Institute, Mansoura Branch, Giza, Egypt; 4 Department of Animal Wealth Development, Faculty of Veterinary Medicine, Mansoura University, Mansoura, Egypt; 5 Department of Pathology, Agricultural Research Center, Animal Health Research Institute, Mansoura Branch, Giza, Egypt; 6 Department of Poultry Diseases, Faculty of Veterinary Medicine, Mansoura University, Mansoura, Egypt; 7 Department of Virology, Reference Laboratory for Veterinary Quality Control On Poultry Production (Gamasa Branch), Agricultural Research Center, Animal Health Research Institute, Giza, Egypt

**Keywords:** RT-PCR, vvIBDV, Genotype classification, Pathogenicity, Reassortment, Chicken farms

## Abstract

This study investigated the genotype classification and pathogenicity of infectious bursal disease virus (IBDV) circulating in vaccinated broiler chicken farms in Egypt. A total of 150 samples were collected from 30 vaccinated commercial broiler chicken farms and pooled into 30 working samples. IBDV was tested using reverse transcriptase polymerase chain reaction (RT-PCR) amplification of the hypervariable region of the viral protein 2 (hvVP2) and the VP1 gene 5’ extremity. Both RT-PCR fragments were sequenced from six samples, and then the obtained nucleotide sequences were analyzed. The IBDV genotypes were identified using nucleotide sequences. Five sequences of the six strains examined were classified as genotype A3B2 for the highly virulent segments A and B (vv-A/vv-B IBDV). Interestingly, this study identified and classified a novel segment-reassortant strain as the A1B2 genotype. Specifically, it involved the segment reassortment of classical virulent segment A (cv-A) with vv-B producing cv-A/vv-B reassortant IBDV. Subsequently, we compared the pathogenicity of reassortant (cv-A/vv-B) IBDV and vvIBDV strains identified in this study. Both strains developed typical IBD clinical signs, postmortem lesions, histopathology, immunohistochemistry, and lesion scores, which were more severe in vvIBDV than reassortant IBDV. In conclusion, this is the first report of the genotype classification based on both genome segments (hvVP2 and VP1) with pathogenicity of IBDV circulating in vaccinated broiler chicken farms and this pathogenicity is more severe in vvIBDV strain than a novel reassortant IBDV strain.

**Supplementary Information:**

The online version contains supplementary material available at 10.1007/s11259-024-10468-z.

## Introduction

The infectious bursal disease virus is a highly infectious and immunosuppressive virus targeting the lymphoid organs of young birds, particularly the bursa of Fabricius (BF). Therefore, this causes the depletion of immature B-lymphocytes and immunosuppression (Alkie and Rautenschlein 2016). IBD is one of the most economically important poultry diseases due to its potential to induce immunosuppression and also strain depended mortality in susceptible chickens. Vaccination failure occurs due to the long-term suppression of the immune system, which in turn allows for the occurrence of infections caused by opportunistic avian pathogens. These infections lead to a decrease in growth performance (Arafat et al. 2017; Jackwood et al. 2018; Eladl et al. 2020). Since the first IBDV report in Egypt in 1974, several re-emergence incidences of vvIBDV have been reported (Metwally et al. 2009; Mosad et al. 2020).

IBD is caused by an *Avibirnavirus*, also known as IBDV, which belongs to the *Birnaviridae* family (ICTV 2023). The bi-segmented genome of IBDV consists of segment A, which is translated into the viral proteins VP0 and VP5. VP0 is subsequently processed into three viral proteins (VP2, VP3, VP4), and segment B, which encodes VP1 (Ebrahimi et al. 2020). IBDV is classified into two serotypes groups based on its distance in antigenicity: serotypes 1 and 2 (Eterradossi and Saif 2020). Based on IBDV serotype 1 virulence, it can be classified into three groups: sub-clinical (scIBDV), classical virulent (cvIBDV), and very virulent (vvIBDV). Antigenically, IBDV serotype 1 is classified into two main groups: variant and classical IBDV, with several subtypes due to antigenic drift (Michel and Jackwood 2017). This IBDV classification is complex and depends on pathotypes and antigenic types. Recently, serotype 1 has been classified into seven main genogroups (vvIBDV, Antigenic variant, Classical, Variant/classical recombinant, ITA, Australian, and dIBDV) depending on phylogenetic analysis of hvVP2 of IBDV strains all over the world (Michel and Jackwood 2017).

Molecular characterization of IBDV strains depending on both genomic segments is highly recommended (OIE 2018). Therefore, a new classification scheme for categorizing IBDV genotypes has been developed by integrating the analysis of sequences of VP2 and VP1 genes. The novel categorization system for IBDV serotype 1 genotypes integrates the sequence analysis of hvVP2 (A1- A9) and VP1 (B1- B5) sequences. It classifies serotype 1 IBDVs into four genotypes: A1B1 (classic IBDV), A2B1 (variant IBDV), A3B2 (vvIBDV), and A8B1 (attenuated IBDV) genotypes (Islam et al. 2021; Zhang et al. 2022; Gao et al. 2023).

The molecular origin of IBDV pathogenicity is not well understood, but some studies have suggested that the changes in amino acid sequences in hvVP2 and VP1 may affect IBDV virulence (Le Nouen et al. 2006; Jackwood et al. 2008, 2018). Given the incomplete understanding of IBD etiology, pathological studies are necessary to investigate the connection between pathogen location and histological alterations and shed light on the pathogenesis of this infection. Further investigation of host–pathogen interactions is necessary to enhance our understanding and control of IBDV, as mutations in the viral genome have altered its antigenicity and virulence (Tanimura 2022).

A few studies have presented the classification of IBDV depending on the sequence analysis of both hvVP2 and VP1 genes (Islam et al. 2021; Wang et al. 2021; Zhang et al. 2022; Gao et al. 2023). There is no published article on this new genotype classification scheme depending on both hvVP2 and VP1 with pathogenicity of IBDV circulating in vaccinated chicken farms. The objective of this study was to identify the molecular nature of IBDV strains circulating in vaccinated commercial broiler chicken flocks depending on the phylogenic analysis of both hvVP2 and VP1 genes besides pathogenicity testing of vvIBDV and reassortant local IBDV strains. Therefore, this study focused on genotype classification and comparative pathogenicity of IBDV circulating in vaccinated broiler chicken farms.

## Materials and methods

### Ethical statement

This study was approved after review by the Mansoura University Animal Care and Use Committee (MU-ACUC), Mansoura University, Egypt, with MU-ACUC (VM.R.23.02.53) code number.

### Samples collection and preparation

A total of 150 clinical samples (bursa of Fabricius (BF), liver, spleen, and kidney) were collected from 30 commercial broiler chicken farms (5 birds/farm). Each farm had previously been vaccinated against IBDV. The farms were located in three governorates in Egypt: Dakahlia, Gharbia, and Damietta. The samples were collected between June 2022 and June 2023. A total of 30 working samples were used in this study, as the samples collected from each individual farm (5 samples in total) were pooled together to create a single working sample. The examined birds were between 15 and 45 days old and were suspected to be infected with IBDV, which exhibited mortality rates ranging from 16 to 35%. The birds were previously vaccinated with different IBDV vaccines, including live attenuated vaccines (Nobilis® Gumboro D78, Nobilis® Gumboro 228E, CEVAC® IBDL, and Bursine® Plus) and live recombinant virus vector vaccines (VAXXITEK® HVT + IBD and INNOVAX-ND-IBD®). The studied birds were vaccinated with different IBDV vaccines either on day 1 or 12 days of age according to farm strategy. Parental stock of these vaccinated chicks were received inactivated IBDV vaccine (Table S1). All samples were collected from farms, which have been operating under the open rearing system with application of some biosecurity measures as washing by disinfectants at the gates, isolation of the sick birds, collection of dead chickens once daily and disinfection of the farm and the equipment before each production cycle. Environmental factors are semi-controlled by heaters in cold weather and fans in hot weather (Mohammed and Helal 2016). From each farm, five morbid or freshly dead birds were collected, and their BF, liver, spleen, and kidney were pooled in one working sample. A negative control sample (pooled BF, liver, spleen, and kidney) was collected from five euthanized apparently healthy 28-day-old non-vaccinated chickens. These samples were homogenized in PBS (10% w/v). Their supernatant fluids were collected after centrifugation at 500 RCF for 15 min and then stored at -20 °C until molecular characterization and isolation of IBDV (Rodriguez-Chavez et al. 2002).

### Molecular identification of IBDV

#### Viral RNA extraction and reverse transcriptase polymerase chain reaction

Viral RNAs were extracted from the supernatant fluids of collected samples according to the instructions of the “QIAamp®MinElut® Virus Spin Kit” (Qiagen GmbH, Germany). Molecular characterization of IBDV in collected field samples was done by RT-PCR partial amplification of the IBDV hvVP2 gene (743 bp) and IBDV VP1 gene 5’ extremity (642 bp) using previously designed primers (Le Nouen et al. 2006; Jackwood et al. 2007) (Table 1). The used primers were sourced from Metabion International AG., Germany. The RT-PCR was applied according to the instructions of the used kit (TOP script TM One-Step RT-PCR kit; enzymotics, Korea). RT-PCR reaction mix composed of one-step RT-PCR mix (5 µl), template RNA (3 µl), and 10 pmol (1 µl) of each primer finally, the DNase/ RNase free water was added up to 20 µl. For RT-PCR amplification, the T-gradient thermal cycler was used (Biometra, Germany). The amplification condition consists of one reverse transcription cycle (50 °C/ 30 min) and 95 °C/ 10 min for reverse transcriptase enzyme inactivation and initial denaturation. The PCR amplification involves a series of cycles (n = 35) consisting of denaturation at 95 °C for 30 s, primer annealing for 30 s at 59 °C for hvVP2 and 64 °C for VP1, and new strand elongation at 72 °C / 1 min. It is followed by a single final elongation cycle at 72 °C /10 min (Le Nouen et al. 2006; Jackwood et al. 2007). The resulting RT-PCR products were electrophoresed in 1.5% agarose gel in TBE buffer (0.5X) containing ethidium bromide at a concentration of 0.5 μg/ml. A Jena Bioscience 100 bp DNA ladder from Germany was also included. The gel was then visualized using a UV transilluminator.

**Table 1:** Details of oligonucleotide primers used in RT-PCR amplification of IBDV VP1 and hvVP2 genes

Primer name	Sequence (5'- 3)	Annealing temp. (^◦^C)	Product size (bp)	reference
VP1-F	TGTAAAACGACGGCCAGTGAATTC-AGATTCTGCAGCCACGGTCTCT	64	642	Le Nouen et al. 2006
VP1-R	CAGGAAACAGCTATGACCCTGCAGTTG-ATGACTTGAGGTTGATTTTG			
hvVP2-F	GCCCAGAGTCTACACCAT	59	743	Jackwood et al. 2007
hvVP2-R	CCCGGATTATGTCTTTGA			

#### DNA sequencing and sequence analysis

The RT-PCR products of the appropriate sizes for both genes (743 bp for the hvVP2 gene and 642 bp for the VP1 gene) were obtained from six RT-PCR positive samples (mans1, mans4, mans5, mans9, mans10, and mans22). The bands with the highest clarity in both genes were carefully excised and purified from the gel using the (QIAquick PCR kit, Qiagen, USA). The purified DNAs obtained were sent to Colors Medical Laboratories in Egypt for bi-directional DNA sequencing. The sequencing was performed using the same primer sets previously used in the RT-PCR. The nucleotide sequences obtained from both genes were submitted to the GenBank database using the Bankit tool of GenBank (NCBI) (http://WWW.ncbi.nlm.nih.gov/Genbank) with accession numbers described in Table 2. In order to detect the molecular nature of the studied IBDV, the obtained nucleotide and deduced amino acid sequences from both genes were analyzed along with other IBDV strains from NCBI, including different vaccine strains used in Egypt. Nobilis® Gumboro D78 (D78 strain), Nobilis® Gumboro 228E (228E strain), CEVAC® IBD L (Winterfield-2512 strain), and Bursine® Plus (Bursine 2 strain) vaccines were included in both hvVP2 and VP1 genes analysis. Only the VAXXITEK® HVT vaccine (Faragher 52 strain) was considered for analysis of the hvVP2 gene, as this vaccine is a recombinant vaccine containing only the IBDV VP2 gene. The INNOVAX-ND-IBD® vaccine was excluded from the sequence analysis due to the manufacturer’s omission of information regarding the specific IBDV strain utilized in the vaccine’s preparation in the product data. Phylogenetic trees were constructed using the maximum-likelihood method with 1000 Bootstrap repeats test in MEGA X software (Kumar et al. 2018). The Clustal-W alignment tool from the Bioedit software was utilized to align the deduced amino acid sequences with other IBDV strains obtained from NCBI (Hall 1999).

**Table 2:** Accession numbers obtained from GenBank for VP1 and hvVP2 genes of identified IBDV strains

IBDV strain	mans1	mans4	man5	mans9	mans10	mans22
Gene						
VP1	OQ883724	OQ883725	OQ883726	OQ883727	OQ883728	OQ883729
hvVP2	OQ030204	OQ030205	OQ030206	OQ030207	OQ030208	OQ030209

### Pathogenicity testing of reassortant (cv-A/vv-B) and vvIBDV strains

#### IBDV isolation in ECEs

Two strains, mans1 (cv-A/vv-B reassortant IBDV) and mans4 (vvIBDV) strains were isolated for three serial passages on the chorioallantoic membrane (CAM) of 12 days old SPF-ECEs (Koum Oshiem, Fayoum, Egypt). Prior to isolation, each sample was initially subjected to a one-hour incubation at room temperature with a combination of broad-spectrum antibiotics and antifungal agents (streptomycin 2 mg/mL, penicillin 2000 units/mL, gentamycin 50 g/mL, and mycostatin 1000 units/mL). The inoculum’s absence of colony forming units was tested by blood agar to indicate it was free from aerobic bacterial and fungal contamination (Islam et al. 2010). Each sample was inoculated in five ECEs (0.2 ml viral suspension per egg), and then inoculated eggs were incubated at 37 °C for 7 days. Dead embryos within the first 24 h post-inoculation (PI) were considered non-specific embryonic death and excluded. Any deaths after 24 h until seven days PI were investigated for embryonic lesions and pock lesions on the CAMs (OIE 2018). The embryos and CAMs were collected from the third egg passage of each strain separately, homogenized, and used for IBDV titration. Moreover, RT-PCR was conducted to confirm the freedom of the tested samples from contamination by Avian influenza viruses (H5 and H9) and Newcastle disease virus (WHO 2005; Monne et al. 2008; El-Morshidy et al. 2021).

#### IBDV titration in ECEs

The IBDV was titrated by preparing a tenfold serial dilution (10^–2^ – 10^–9^) from each IBDV strain. Subsequently, 0.2 ml from each dilution was applied to the CAM of 12-day-old SPF-ECEs (5 eggs/ dilution). The IBDV titer was determined as the 50% embryo infective dose per ml (EID_50_/ml), following the method previously described by Reed and Muench (1938). The virus titers were 10^6.5^ EID_50_/ml for cv-A/vv-B reassortant IBDV (mans1) and 10^6^ EID_50_/ml for vvIBDV (mans4).

#### Pathogenicity test

Thirty SPF-ECEs (Koum Oshiem, Fayoum, Egypt) were incubated aseptically until hatching. The hatched chicks (n = 30) were bred in a well-ventilated isolated room and fed ad libitum with a commercial starter diet (Dakahlia Poultry Company, Egypt) from 1st to the 21st day of age followed by a grower diet from the 22nd till the end of the experiment without any medication or vaccination. On day 21, the chicks were divided into three groups of ten birds (G1, G2 and G3). Birds in G1 were uninoculated control birds; G2 birds were experimentally infected with cv-A/vv-B reassortant IBDV (mans1), while G3 birds were infected with vvIBDV strain (mans4). At the 21st day of age, G2 and G3 birds were inoculated with 0.1 ml IBDV by oculo-nasal route (10^5^ EID_50_/0.1 ml). The G1 birds were kept without inoculation (negative control group) with physical separation of experimental groups. The pathogenicity of cv-A/vv-B reassortant IBDV and vvIBDV was assessed by mortality percentage, clinical signs, postmortem lesions, histopathological examination, bursal lesion score, immunohistochemistry, virus shedding and IBDV antibody detection. Five days PI, three birds were taken randomly from each group and then euthanized. A part from BF was collected and used to confirm IBDV infection by RT-PCR amplification of the IBDV VP1 gene, as previously described by Le Nouen et al. (2006). Spleen, thymus, kidney, and the other part of BF were collected from euthanized birds, preserved in 10% neutral formalin, and then used for histopathological studies. The formalin-preserved specimens were then immersed in different alcohol concentrations, embedded in paraffin, cut with a microtome at 4-μm thickness, and stained by haematoxylin and eosin (H&E). The stained tissues were microscopically examined for the presence of lesions (Bancroft et al. 1996). The stained BF, spleen, thymus, and kidney tissue sections were studied under a light microscope. The lesions were documented and evaluated using the following scoring system: The lymphoid follicles are classified into five categories based on their level of depletion: 0 for apparently normal follicles, 1 for mild depletion, 2 for moderate depletion, 3 for severe depletion, and 4 for follicle atrophy with or without cystic spaces (Raue et al. 2004). The immunohistochemical examination was performed to detect intracellular IBDV antigen in formalin-fixed, paraffin-embedded bursa, spleen, thymus, and kidney. This test was performed at the Animal Health Research Institute, Dokki, Giza, Egypt. In this test, the avidin–biotin complex immunoperoxidase method with a commercially available test kit (Rabbit-specific HRP/DAB (ABC) IHC detection Kit, ab64261, Abcam, UK) was used for the detection of viral antigen according to manufacturer’s directions. Sections (4 μm) were cut, mounted on coated glass slides, and left to dry overnight. After dewaxing and dehydration, blocking steps in the procedure, including incubation with 0.3% hydrogen peroxide, were applied. The monoclonal antibodies against IBDV (kindly provided by RLQP, Animal Health Research Institute) were diluted in 1:500 with diluting buffer prior to application to the sections. The antigen was visualized using a substrate solution containing 0.5% 3, 3-diaminobenzidine (DAB). The sections were counterstained with Mayer’s haematoxylin. RT-PCR was also used to identify IBDV shedding in faecal samples collected at the 5th and 7th days PI. The chickens were observed for up to 7 days PI for mortality and clinical signs and then euthanized for postmortem (PM) examination. On the 7th day PI, serum samples were collected for ELISA testing to evaluate the production of IBDV antibodies in infected chickens as part of the experiment. The IBDV antibody titers were measured using the Biocheck ELISA kit (Smart Veterinary Diagnostics, Netherlands) (Singh et al. 2010).

#### Statistical analysis

The data were subjected to statistical analysis to determine their significance using an unpaired two-tailed Student’s t-test. *P* value < 0.05 was considered significant (Murmu et al. 2014).

## Results

### Clinical signs and PM lesions of naturally infected chicken farms

Examination of chicken farms revealed the distinct mortality pattern associated with IBDV. The mortality curve displayed a rapid increase in mortality for a period of 5–7 days from clinical signs appearance, followed by a sudden decline. The highest mortality rate was observed on the 3rd day from clinical signs appearance. The chickens exhibited symptoms of anorexia, dehydration, excessive water intake, ruffled feathers, recumbency, whitish watery diarrhea, swollen, soiled vents, trembling, and prostration. The postmortem examination revealed inflammation, edema, and haemorrhagic BF in some birds and atrophied in others. Muscle dehydration was also observed with haemorrhages in the pectoral and thigh muscles. Kidneys were enlarged and haemorrhagic with urate deposition.

### Molecular identification of IBDV

#### Reverse transcriptase polymerase chain reaction

Out of 30 tested samples, 21 showed positive RT-PCR results for both hvVP2 (743 bp) and VP1 (642 bp) genes. Conversely, the remaining nine samples were negative for both genes (Table S1).

#### DNA sequencing and sequence analysis

**Fig. 1:**
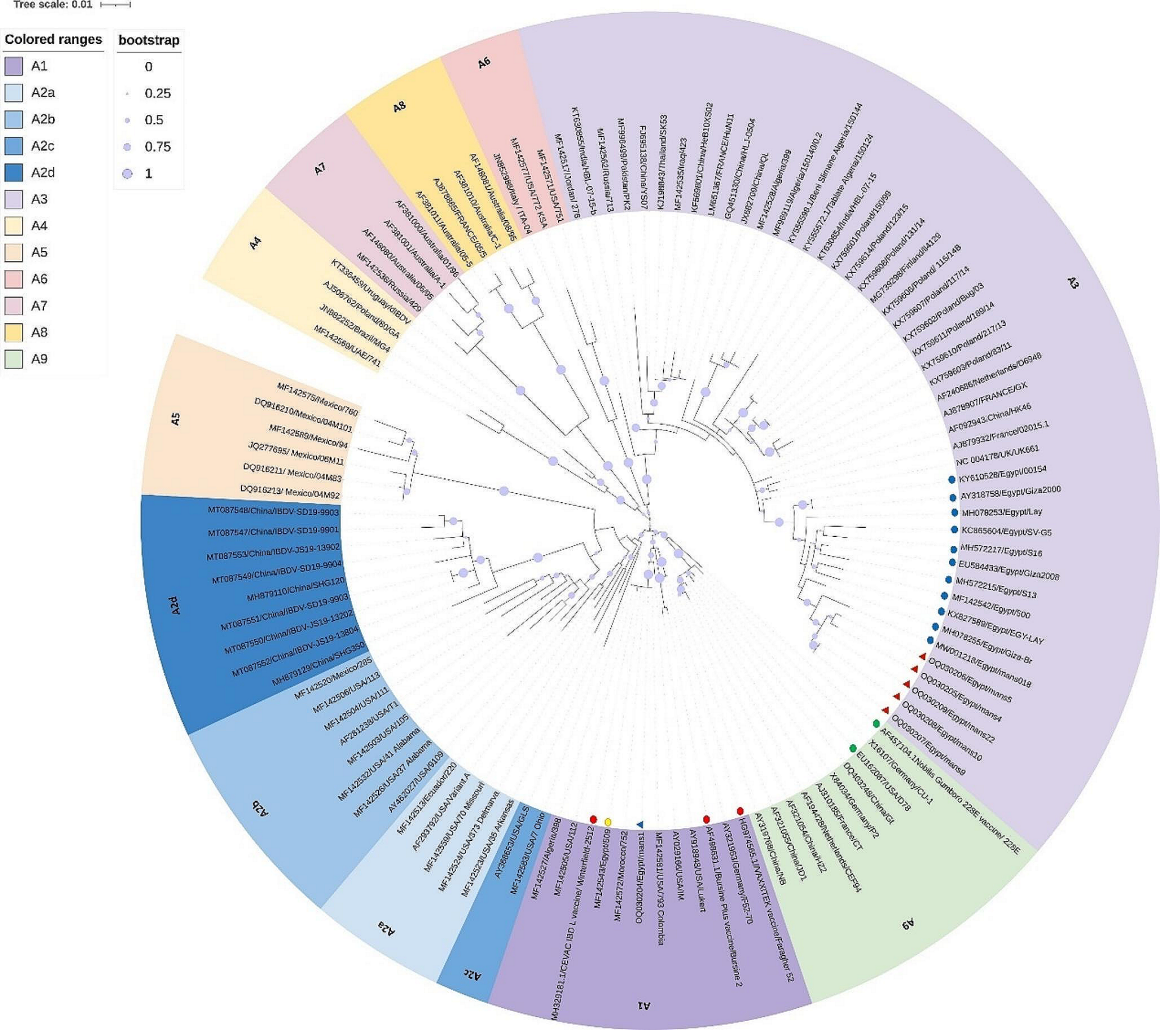
Phylogenetic tree (Maximum-likelihood) constructed on the nucleotide sequences of hvVP2 gene, which divided into nine major clades (nine genogroups). The nine major genogroups are named A1- A9 according to Gao et al. (2023). Our strains (red triangles) were clustered in genogroup A3 (vvIBDVs) with other Egyptian strains (Blue dots). While the mans1 strain (blue triangle) was grouped in genogroup A1 together with other classic IBDV including the Egyptian “509” classic IBDV strain (yellow dot) and vaccine strains (red dots). Other vaccines were grouped in genogroup A9 together with other attenuated IBDV (green dots)

Six RT-PCR positive samples (mans1, mans4, mans5, mans9, mans10 and mans22) which showed the sharpest bands in both hvVP2 (743 bp) and VP1 (642 bp) were selected (Table S1). The DNA of both genes from these six samples was extracted from the gel and sequenced. The maximum-likelihood phylogenetic tree based on the hvVP2 gene nucleotide sequences of six sequenced samples and other IBDV serotype I sequences from NCBI was divided into nine major clades (nine genogroups). The nine major genogroups are named A1-A9, as per Gao et al. (2023). Moreover, the genogroup A2 was divided into four subgroups (A2a, b, c, d). According to Michel and Jackwood (2017) and Gao et al. (2023) IBDV classification, out of six strains identified in this study, five strains (mans4, mans5, mans9, mans10, and mans22) were clustered in genogroup A3. This genogroup is composed of vvIBDV strains. The five strains were closely related to other Egyptian vvIBDVs and aligned in the same subclade. In contrast, the other strain, mans1, was grouped in genogroup A1 together with other classic IBDV, including the Egyptian 509 classic IBDV strain and three vaccine strains used in Egypt (CEVAC® IBDL, Bursine® Plus and VAXXITEK® HVT + IBD). In addition, the other two vaccine strains (Nobilis® Gumboro D78 and Nobilis® Gumboro 228E) were clustered in the new genogroup A9 of attenuated IBDV strains (Fig. 1).

**Fig. 2:**
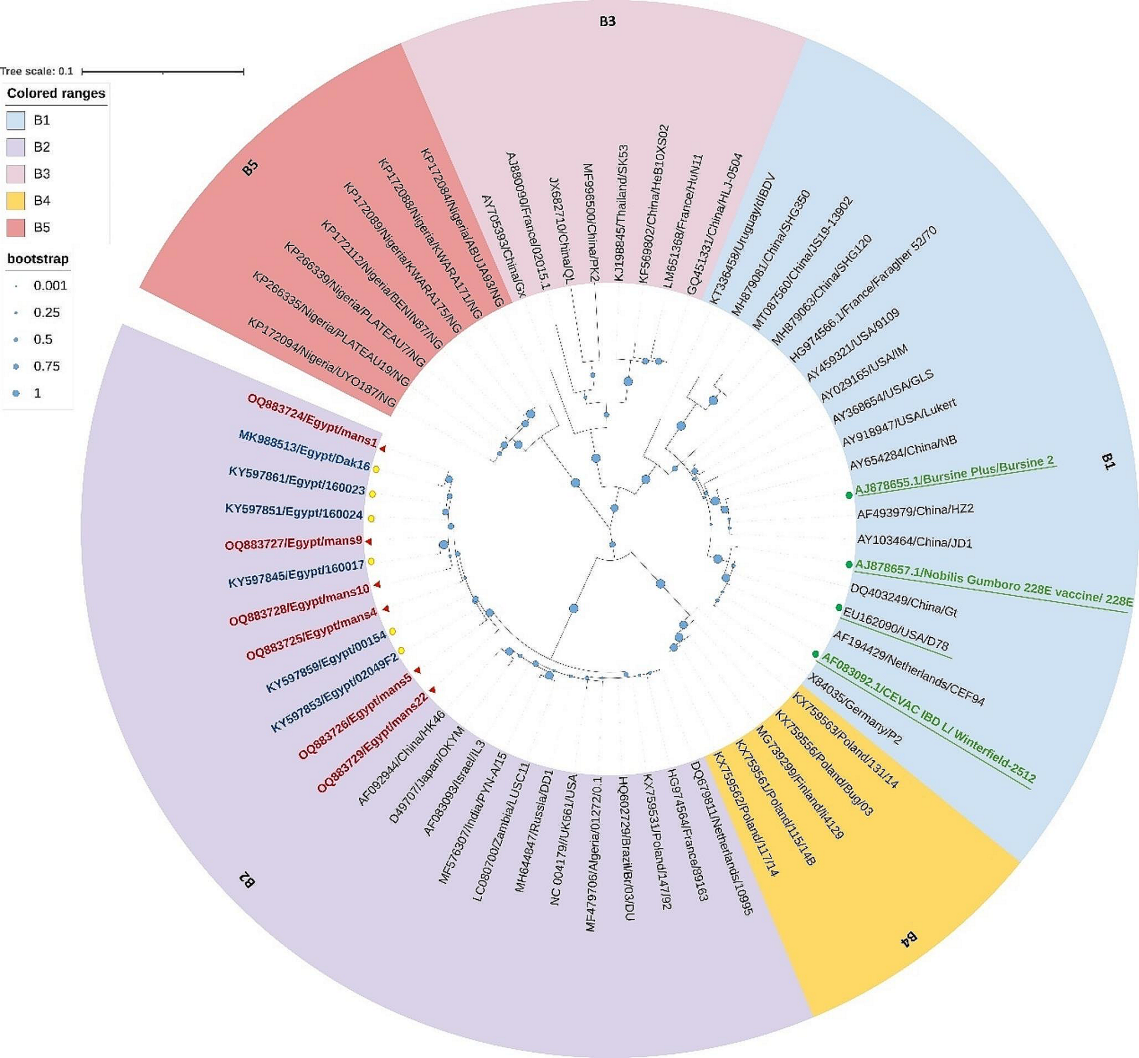
Phylogenetic tree (Maximum-likelihood) established on the nucleotide sequences of VP1 gene, which divided into five major clades (five genogroups). The five major genogroups are named B1- B5 according to Gao et al. (2023). Our strains (red triangles) were clustered in genogroup B2 (vvIBDVs) with other Egyptian strains (yellow dots) in a separate subclade. While all vaccine strains were clustered in genogroup B1 (green dots)

The maximum-likelihood phylogenetic tree constructed on the nucleotide sequences of the VP1 gene from six sequenced samples and other IBDV serotype I sequences from NCBI was divided into five major clades (five genogroups). The five main genogroups were named B1-B5. The six strains we observed were grouped together in genogroup B2, which consists of vvIBDV strains as classified according to Gao et al. (2023) IBDV classification. Strains in our study were clustered with other Egyptian vvIBDV strains in a separate subclade. The vaccine strains (Nobilis® Gumboro D78, Nobilis® Gumboro 228E, CEVAC® IBDL, and Bursine® Plus) were grouped in genogroup B1 together with early variant, attenuated, classic strains, Chinese novel variant strains and Algerian reassortant strains (Fan et al. 2020) (Fig. 2). According to Gao et al. (2023) advanced IBDV genotype classification depending on the combining of both hvVP2 and VP1 genes sequence characteristics, five strains (mans4, mans5, mans9, mans10 and mans22) were classified in genotype A3B2 (vvIBDV). Interestingly, a novel segment-reassortant strain detected in our study (mans1) was classified into genotype A1B2 in which segment-reassortment of classical virulent segment A (cv-A) with very virulent segment B (vv-B) producing cv-A/vv-B reassortant IBDV.

BioEdit software was used to analyze both hvVP2 and VP1 genes deduced amino acid sequences from our strains and other reference strains from GenBank. Deduced amino acid sequences of the hvVP2 gene (206- 350 aa) revealed the presence of vvIBD amino acid signature in five strains (mans4, mans5, mans9, mans10, and mans22) which were classified in genotype A3B2. The virulence amino acids signature (222A, 242I, 249Q, 253Q, S254, 256I, G258, T260, T269, A270, 272I, A278, 279D, 284A, 286 T, 294I, 299S, 318G, A321, 324L, and S330) were identified. The characteristic serine-rich heptapeptide sequence SWSASGS of vvIBDVs (326- 323aa) was also identified in all aligned sequences.

**Fig. 3:**
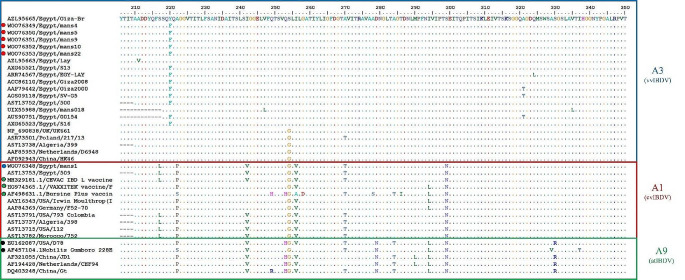
BioEdit analysis of deduced amino acid sequences of hvVP2 gene (206- 350 aa) with Giza-Br strain as a reference strain. The alignment revealed the presence of A3 vvIBD amino acid signature in five strains (Red dots). Meanwhile, the mans1 strain (Blue dot) showed the same amino acid profile of A1 classical virulent IBDVs including vaccine strains (green dots). Other vaccine strains A9 (attenuated IBDV) included other vaccine strains (black dots)

There were significant conserved amino acid substitutions at the surface P_BC_ loop (Y220F) in most Egyptian strains and the P_DE_ loop (G254S) in all Egyptian strains. Strain mans1 in our study was classified with classical virulent IBDVs together with CEVAC® IBD L, Bursine® Plus, and VAXXITEK® HVT + IBD vaccines. The majority of cvIBDVs exhibited conserved amino acid substitutions as (S217L, A222P, I242V, I256V, A270T, N293L, and S299N) when compared to vvIBDV strains (Fig. 3). These amino acid substitutions were also detected in related vaccines (CEVAC® IBD L, Bursine® Plus and VAXXITEK® HVT + IBD). These amino acid substitutions were also recorded in attenuated IBDV strains aligned in the new genogroup A9, except the amino acid substitution S217L detected in most A1 cvIBDVs, not A9 atIBDVs. The genogroup A9 viruses have extra conserved amino acid substitutions (Q253H, D279N, A284T, and S330R), which were not detected in A1 or A3 viruses. The cvIBDV strain (mans1) in this study was found to be closely related to CEVAC® IBDL vaccine, as they shared the same amino acid profile with 100% amino acid identity (0% diversity). In contrast, the Bursine® Plus vaccine contains specific distinct amino acid substitutions that are not found in other vaccines or strains belonging to the same genogroup. Consequently, it is the most distinct vaccine from the mans1 strain (7.59% diversity; Fig. 3 & Table S1).

**Fig. 4:**
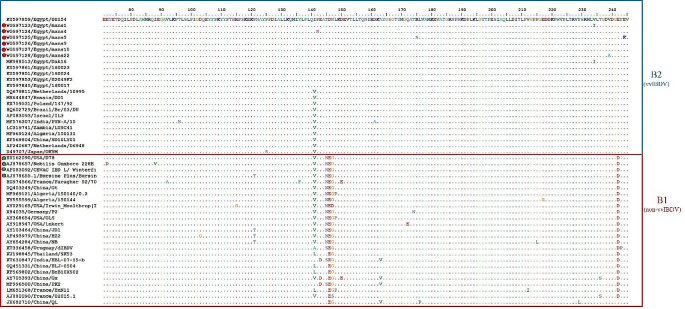
VP1 gene deduced amino acid sequences (71- 245 aa) analysis by BioEdit software with Egypt 00154 as a reference strain. The alignment revealed the presence of vvIBD amino acid signature in all studied strains (Red dots). Egyptian vvIBDVs have a unique amino acid mutation “V141I”. Vaccine strains (green dots) have non-vvIBDV amino acid signature

The VP1 gene is typically classified into two genogroups: B1 (non-vvIBDV) and B2 (vvIBDV). The B3 genogroup consists of HLJ0504-like strains, the B4 genogroup includes European transitional strains, and the B5 genogroup comprises Nigerian IBDVs. The amino acid sequences (71–245 aa) of the VP1 gene were aligned using BioEdit software. The alignment results showed that our six sequences (mans1, mans4, mans5, mans9, mans10, and mans22) were similar to the vvIBDV pathotype (genogroup B2). All genogroup B2 vvIBDVs have the characteristic tripeptide (TDN) at position 145-147aa, whereas the non-vvIBD (genogroup B1) has (N/SEG) at the same position. Some amino acid substitutions (T145N, D146E, N147G, and E246D) were identified in genogroup B1 (non-vvIBDVs), including CEVAC® IBD L, Bursine® Plus, Nobilis® Gumboro D78, and Nobilis® Gumboro 228E vaccines. The Egyptian genotype B1 vvIBDVs has the unique amino acid mutation V141I (Fig. 4).

### Pathogenicity testing of reassortant (cv-A/vv-B) and vvIBDV strains

#### IBDV isolation in ECEs

**Fig. 5:**
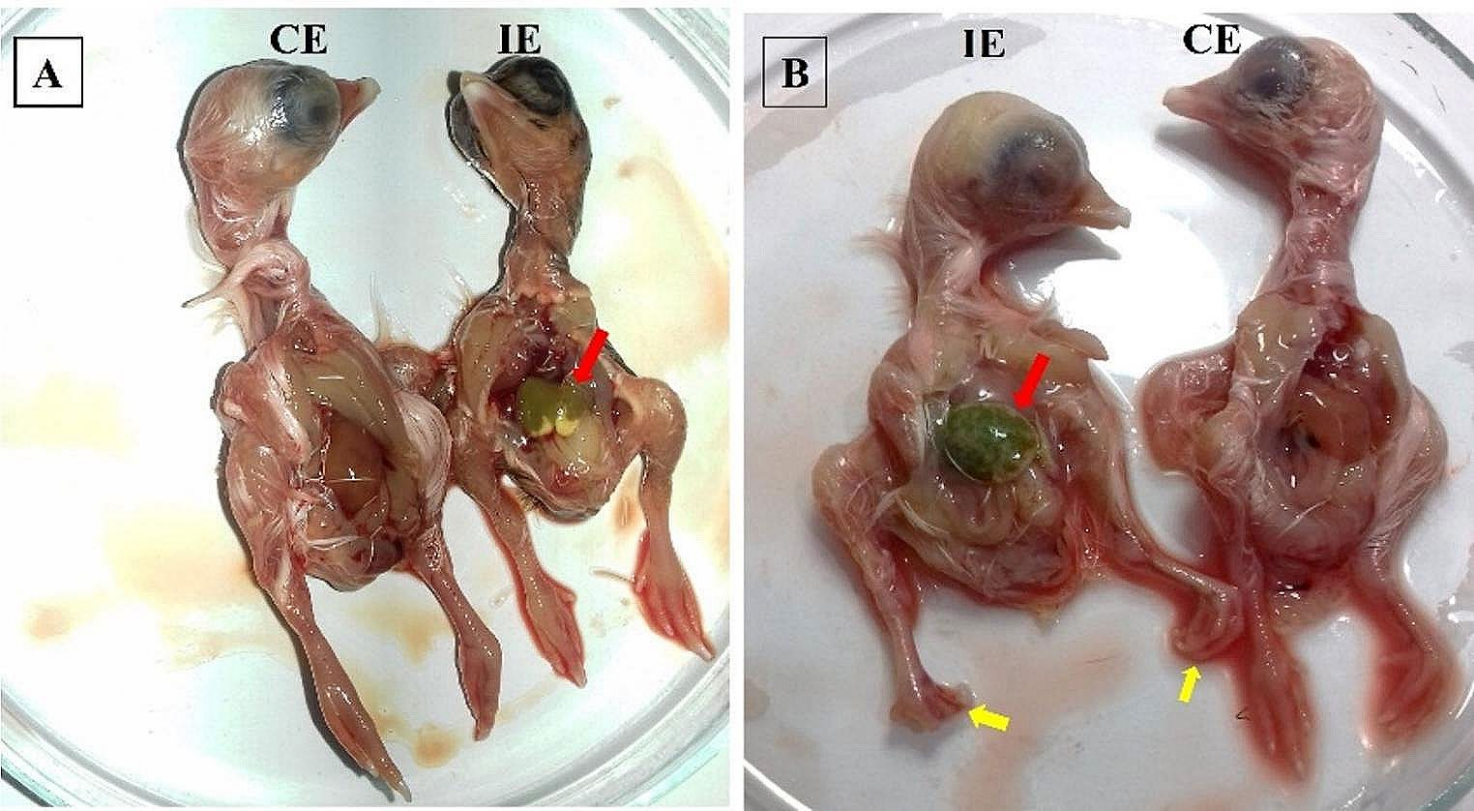
Embryoic lesions of IBDV isolation in SPF-ECEs. (**A**) Lesions of cv-A/vv-B IBDV inoculation in 12 days old ECEs 5 days PI: (CE) non-infected control embryo. (IE) cv-A/vv-B IBDV inoculated embryo showing stunted growth, abnormal feathering, and greenish pale liver with pale outlines (red arrow). (**B**) Lesions of vvIBDV inoculation in 12 days old ECEs 5 days PI: (CE) non-infected control embryo. (IE) embryo inoculated with vvIBDV, showing dwarfism, swollen pale greenish liver (red arrow) with leg deformities (yellow arrows)

The cv-A/vv-B reassortant IBDV (mans1) and vvIBDV (mans4) strains were isolated for three serial passages on the CAM of 12-day-old SPF-ECEs. Both samples showed characteristic embryonic changes during the three passages in SPF eggs. The embryonic lesions included stunted growth, edema, and abdominal distention, with subcutaneous ecchymotic hemorrhages. The CAMs were thickened and congested CAMs. The liver of analyzed embryos obtained from inoculated SPF eggs was greenish, pale, and swollen. The embryonic death occurred in 3–5 days PI. Leg deformities were observed in vvIBDV inoculated embryos (Fig. 5).

#### Pathogenicity test

**Fig. 6:**
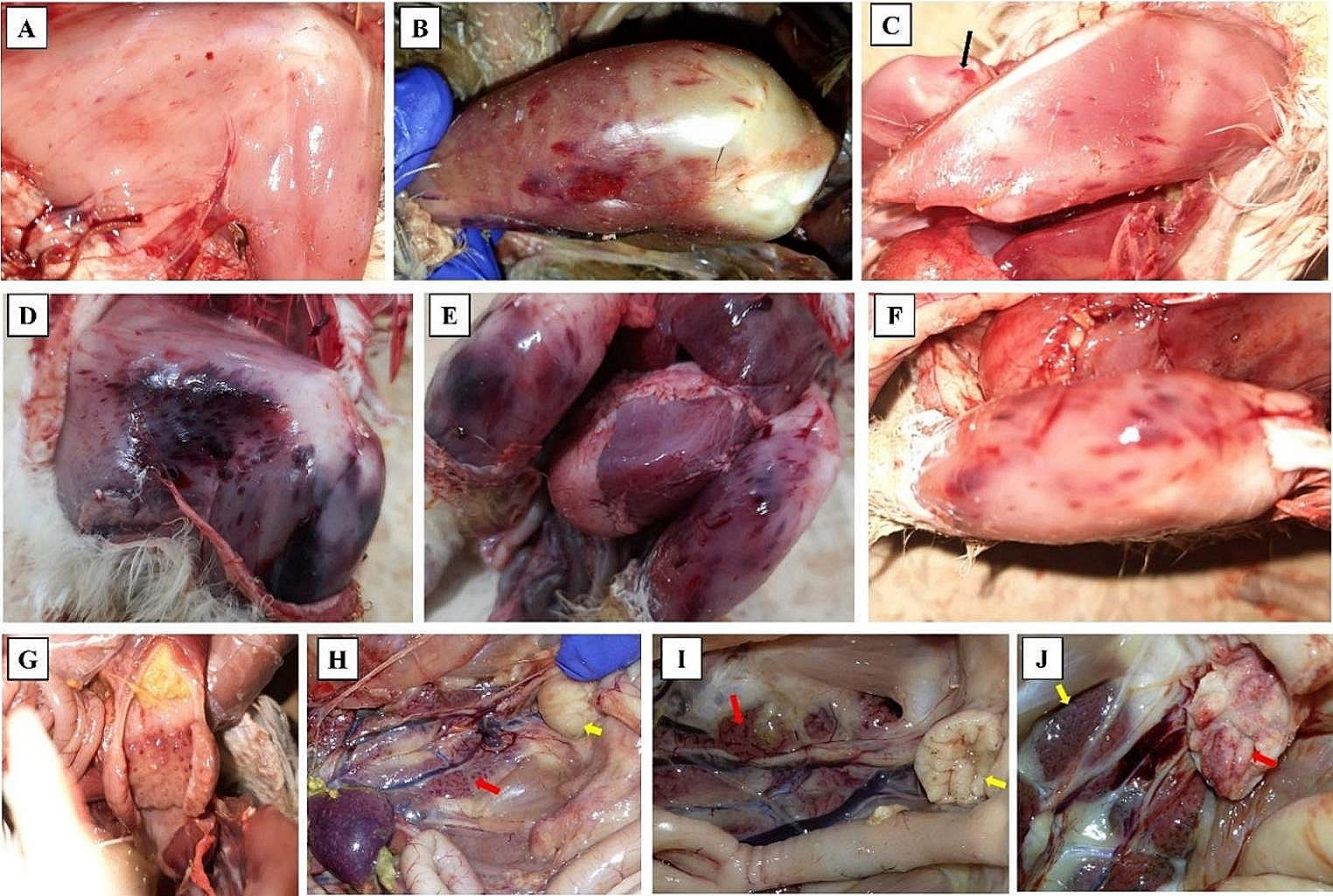
Postmortem lesions of IBDV experimentally infected chickens. **A**; **B**; and **C**: hemorrhages on thigh and pectoral muscles of chickens infected with cv-A/vv-B reassortant IBDV. **D**; **E** and **F**: severe hemorrhages on thigh muscles of chickens infected with vvIBDV. **G**: hemorrhages on the proventricular glands in vvIBDV infected chicken. **H**: vvIBDV infected chicken showing enlarged mottled spleen, enlarged BF (yellow arrow) and nephritis (Red arrow). **I**: cv-A/vv-B reassortant IBDV infected chicken showing nephritis (Red arrow) and swollen BF with gelatinous exudate (yellow arrow). **J**: vvIBDV infected chicken showing nephritis (yellow arrow) and swollen hemorrhagic BF with gelatinous exudates

G1, the non-infected control group, did not exhibit any clinical signs, postmortem lesions, or mortality throughout the entire 7-day experimental period. In contrast, the birds in G2 (cv-A/vv-B reassortant IBDV) and G3 (vvIBDV) exhibited clinical signs such as ruffled feathers, anorexia, whitish diarrhea, depression, and soiled vent (these signs started at the second day PI until the 7th days PI). The postmortem lesions in G2 and G3 included ecchymotic hemorrhages in the thigh and pectoral muscles of G2 birds (Fig. 6A, B, and C). More severe hemorrhages were observed in the thigh and pectoral muscles of G3 birds (Fig. 6D, E, and F). Hemorrhages on the proventricular glands were also observed in G3 birds (Fig. 6G). An enlarged mottled spleen was observed in some vvIBDV-infected birds (Fig. 6H). Swollen hemorrhagic BF with gelatinous exudates and nephritis with urates deposition in ureters (Fig. 6I and J) were also observed. The mortality rate was 60% (6/10) in cv-A/vv-B reassortant IBDV-inoculated chickens (G2) and 70% (7/10) in vvIBDV-inoculated chickens (G3). The IBDV was confirmed in infected chickens of G2 and G3 by RT-PCR amplification of the partial VP1 gene 5 days PI. RT-PCR amplification of the partial IBDV-VP1 gene from fecal samples confirmed that the IBDV-infected chickens shed the virus in their feces on the 5th and 7th days PI in both G2 and G3. However, no IBDV shedding was found in G1 (the control group). IBDV antibodies were evaluated on the 7th day post-infection. The ELISA-tested sera from the inoculated chickens in groups G2 and G3 were positive, with geometric mean antibody titers of 6885 and 6763, respectively (Table S1). Conversely, sera from chickens in the G1 group were negative.

#### Histopathology, immunohistochemical staining and lesion scores

**Fig. 7:**
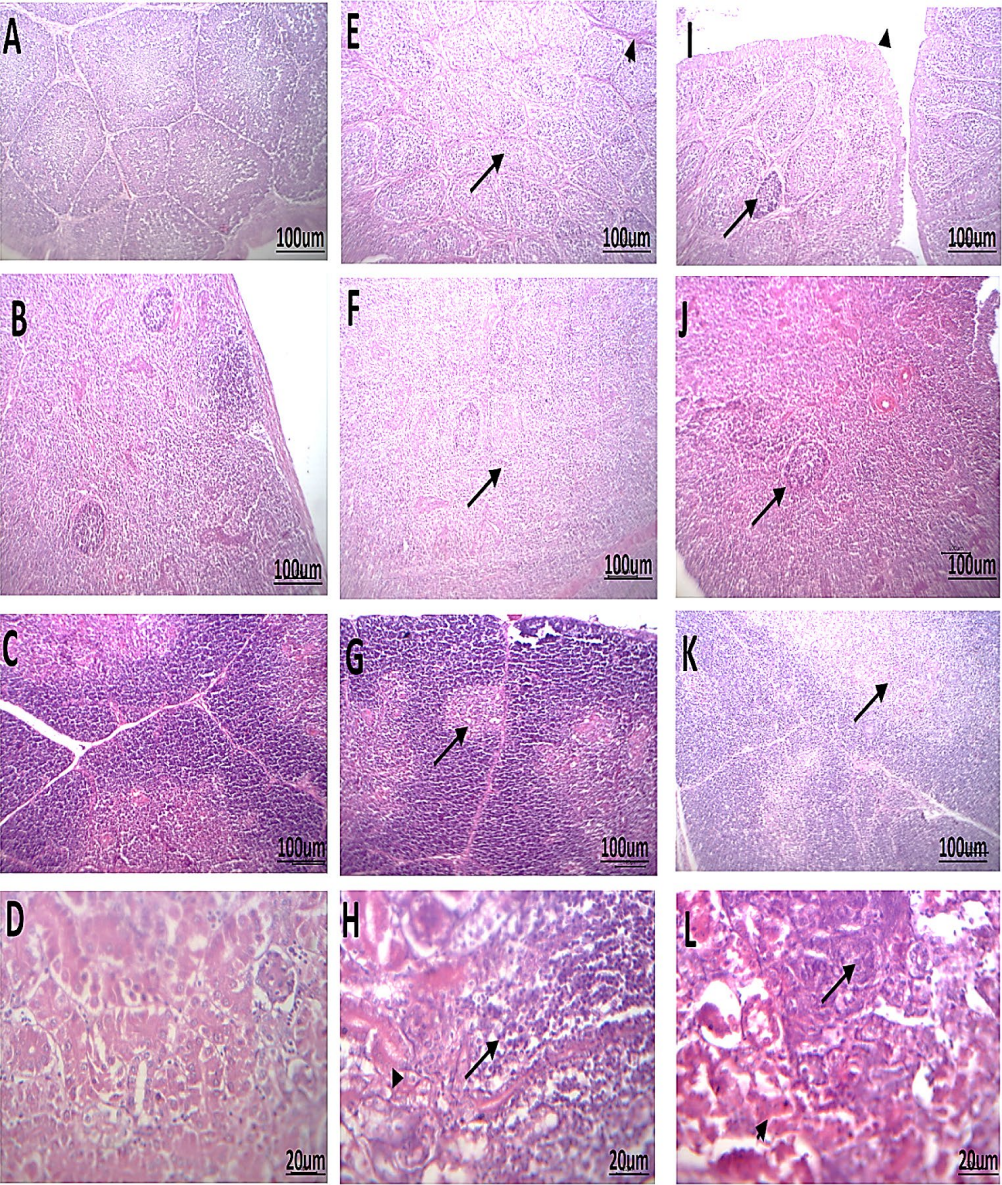
Photomicrograph of sections from bursa of Fabricius, spleen, thymus and kidney of chicken infected with IBD stained with H&E X400. (**A**) Histological picture of bursa of Fabricius shows normal picture in G1 (Control group); (**B**) normal splenic tissue in G1; (**C**) Normal cortex and medulla of thymic lobule in G1; (**D**) Normal renal parenchyma of nephrons; (**E**) Lymphoid depletion of both cortex and medulla (arrow) and interfollicular fibrous tissue proliferation (arrow head) of bursa in G2; (F) Lymphoid depletion from white and red pulps (arrow) of spleen in G2; (**G**) Focal area of lymphoid depletion in cortex of thymic lobule (arrow) in G2; (**H**) Lymphatic aggregates in intersitium (arrow) and necrotic tubules in adjacent parenchyma (arrow head) of kidney in G2; (**I**) Hyperplastic follicles (arrow) and degenerated epithelia with vesicles formation in covering epithelium (arrow head) of bursa in G3; (**J**) Hyperplastic white pulps (arrow) of spleen in G3; (**K**) Mild lymphoid depletion from medulla and replaced by edema (arrow) of thymic lobule in G3; (**L**) Regenerative attempts from renal tubule epithelia (arrow) and nephrotic tubules in adjacent parenchyma (arrow head) of kidney in G3

Photomicrograph of sections from the bursa of Fabricius, spleen, thymus, and kidney of chickens infected with IBDV was shown (Fig. 7). Upon histopathological examination of G1 non-infected chickens, the BF displayed a normal picture (Fig. 7A). However, G2 of cv-A/vv-B reassortant IBDV-infected chickens revealed hyperplastic follicles and degenerated epithelia with vesicles formation in covering epithelium of bursa (Fig. 7E). Whereas, the G3 of vvIBDV infected chickens showed lymphoid depletion of both cortex and medulla and interfollicular fibrous tissue proliferation of bursa of Fabricius (Fig. 7I).

**Fig. 8:**
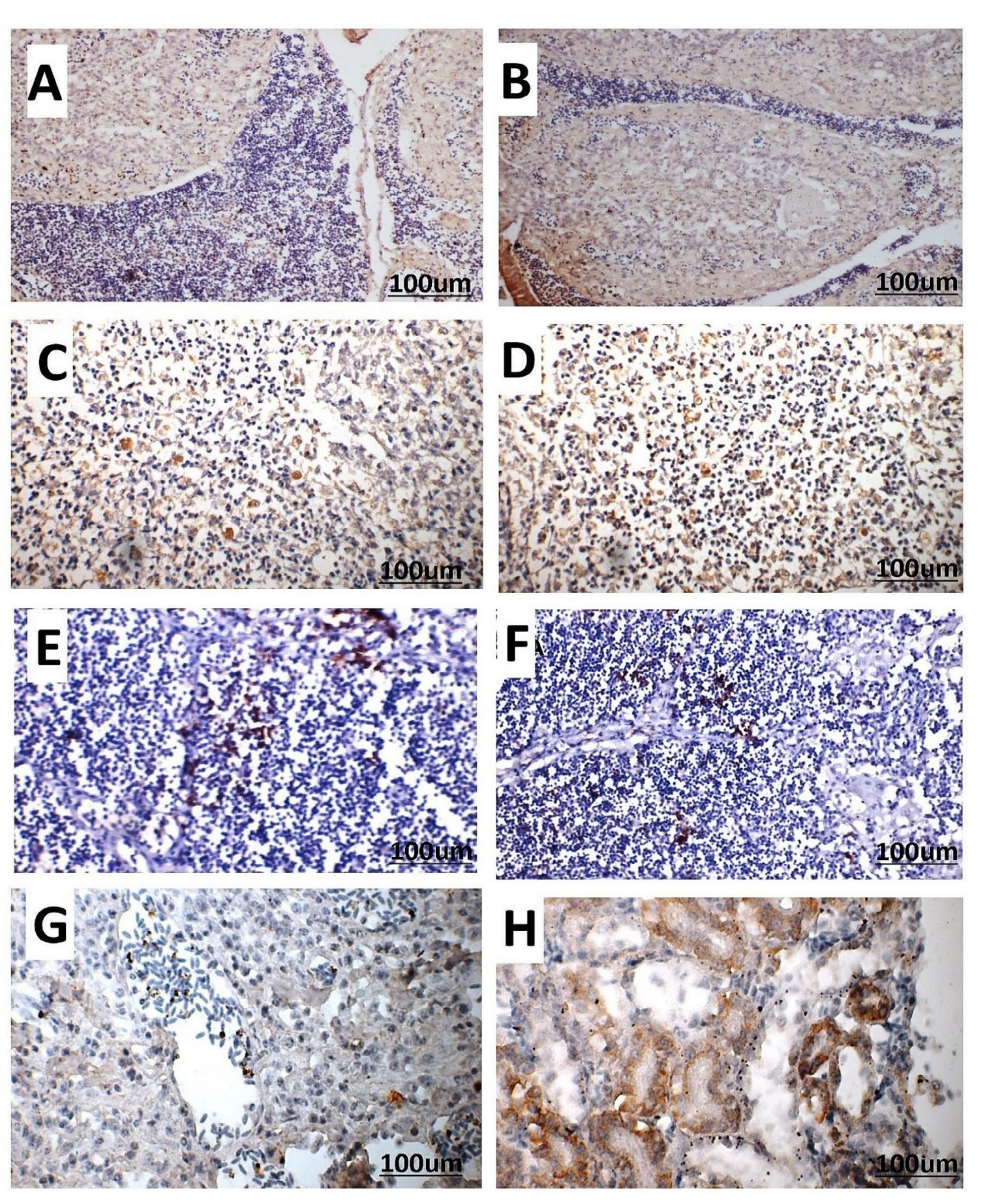
Immunohistochemistry of experimental infected chickens with IBDV, avidin biotin counter stain with Mayer’s haematoxylin (× 400). (**A**) Bursa shows moderate positive immunolabeling of IBDV antigen by immunoperoxidase method in the follicular medulla in G2. (**B**) Bursa shows marked positive immunolabeling of IBDV antigen by immunoperoxidase method in the follicular lymphocytes and interfollicular septa in G3. (**C**) Spleen shows moderate positive immunolabeling of IBDV antigen in some lymphoid cells in both white and red pulps in G2. (**D**) Spleen shows marked positive immunolabeling of IBDV antigen in some lymphoid cells in both white and red pulps in G3. (**E**) Thymus shows positive immunolabeling of IBDV antigen in some lymphocytes in G2. (**F**) Thymus shows positive immunolabeling of IBDV antigen in some lymphocytes in G3. (**G**) Kidney shows positive immunolabeling of IBDV antigen in some lining tubular epithelium in G2. (**H**) Kidney shows marked positive immunolabeling of IBDV antigen in some lining tubular epithelium in G3

**Fig. 9:**
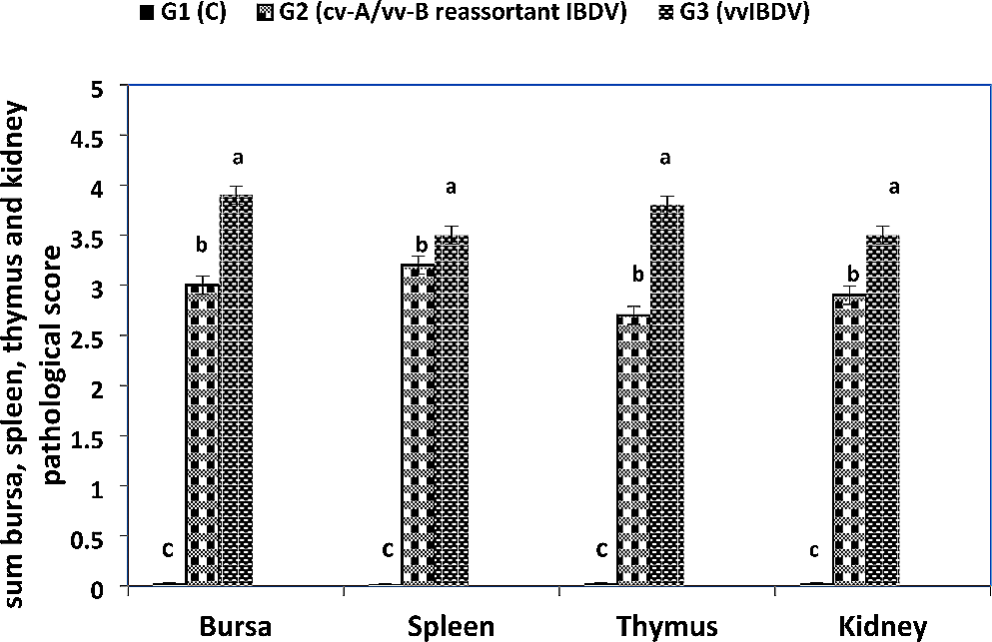
Statistical analysis of lesions in bursa, spleen, thymus and kidney. C, control; vvIBDV, very virulent IBVD; G1, control; G2, infected with cv-A/vv-B reassortant IBDV; G3, infected with vvIBDV. Statistical analysis showed a higher of total bursa, thymus and kidney lesion score in vvIBDV than cv-A/vv-B reassortant IBDV-infected chickens. Different small alphabetical letters mean significant when *P* < 0.05

Using monoclonal antibody, viral antigen of IBDV was detected in the bursa inside lymphocyte of lymphoid follicles and interfollicular septa. Viral antigen was detected in the red and white pulps and thymus lymphocytes. Moreover, it was detected in the renal lining tubular epithelium. All sections were tested negative for avian influenza virus and Newcastle disease virus antigens for their exclusion (Fig. 8). Based on the histopathological study, the lesion scores of experimental chickens revealed that vvIBDV-infected chickens demonstrated higher lesion score than cv-A/vv-B reassortant IBDV-infected chickens (Fig. 9).

## Discussion

Infectious bursal disease is a highly infectious and economically important poultry disease that causes immunosuppression. Despite efforts to control it through vaccination, frequent outbreaks of IBD has been observed worldwide. The nature of IBDV complicates control efforts due to its bi-segmented dsRNA genome, which undergoes frequent genetic mutations and reassortment of genome segments. This leads to changes in antigenicity and the evolution of more virulent strains (Jackwood et al. 2008; Jackwood and Sommer-Wagner 2011). Continuous identification and monitoring of the molecular nature of the circulating IBDV is crucial for providing sufficient data for local vaccine strain development in localities where strict management and repeated vaccination is not sufficient for disease control. Therefore, this study investigated the genotype classification and pathogenicity of IBDV circulating in vaccinated broiler chicken farms in Egypt.

In this study, 150 clinical samples were gathered from 30 commercial IBDV-vaccinated broiler chicken farms. The studied birds were 15–45 days old with 16–35% mortality rates with typical IBDV signs and PM lesions. These findings are consistent with Wagari (2018) and Almaw et al. (2022), who detected 16.7% -30% mortality in broilers infected with vvIBDV. Based on the molecular characterization of IBDV, Petkov et al. (2007) emphasized the significance of sequencing the entire genome of IBDV for molecular characterization. However, this approach is expensive and not feasible when studying a large number of isolates. Most researchers primarily focus on sequencing the hvVP2 region (amino acids 206–350), as it determines the antigenic phenotype and serotype of IBDV (Omar et al. 2021). The hvVP2 is regarded as the antigen that induces the protective immunity, the main factor that determines virulence, and the determinant of the antigenic phenotype, specifically the amino acids located at the apex of the loop structures, including P_BC_, P_DE_, P_FG_, and P_HI_. Single point mutations in these loop structures have been shown to enhance IBDV antigenic drift, thereby compromising the efficacy of the currently used IBD vaccines (Coulibaly et al. 2005; Jackwood and Sommer-Wagner 2011; Michel and Jackwood 2017). Although the hvVP2 plays a crucial role in IBDV genetic evolution, it is not adequate for the detection of all IBDV genogroups. VP1 also has a significant impact on IBDV virulence, emergence, and genetic evolution (Pikuła et al. 2020; Jackwood et al. 2011).

For the classification of segmented viruses, it is more logical to study all appropriate genes, so the molecular identification of both genomic segments is vital as different IBDV reassortants with modified pathogenicity have been increasingly described (Le Nouen et al. 2006; Jackwood et al. 2011; Pikuła et al. 2018, 2020). This study classified six strains of IBDV (mans1, mans4, mans5, mans9, mans10, and mans22) based on the sequence characteristics of both hvVP2 and the partial VP1 coding sequence. The phylogenetic analysis of both genes revealed that five strains (mans4, mans5, mans9, mans10 and mans22) were classified in genotype A3B2 (vvIBDVs). An important evolutionary feature of IBDV is the segment reassortment within circulating strains. The significance of this phenomenon should not be ignored, as it amplifies the difficulty of controlling IBDV and increases epidemiological complexity (Zhang et al. 2022). Interestingly, a novel cv-A/vv-B reassortant IBDV strain was detected in our study (mans1), which was classified into genotype A1B2 according to the IBDV classification of Michel and Jackwood (2017) and Gao et al. (2023). Other reassortants were previously described by Pikuła et al. (2020), who detected a reassortant IBDV strain containing vvIBDV segment A and segment B from an unidentified source. Lu et al. (2015) detected A3B1 (vv-A/at-B) reassortant IBDV. This strain has also been reported in other countries, including Venezuela (Le Nouen et al. 2006), Korea (Lee et al. 2017), and Poland (Pikuła et al. 2018). Another A8B2 (at-A/vv-B) segment-reassortant IBDV was also detected (Cui et al. 2013). About 83% of our studied strains (5/6) are vvIBDV, and this finding is supported by Jackwood and Sommer-Wagner (2007) and Dey et al. (2019), who reported that 60–76% of the worldwide IBDV isolates are vvIBDV genotype. In addition, Yilmaz et al. (2019) reported that 90.5% of their studied IBDV strains were classical strains.

In the present study, the hvVP2-deduced amino acid sequences have vvIBD amino acid signature with the characteristic serine-rich heptapeptide (326-323aa) sequence SWSASGS of vvIBDVs, and this result was supported by Akhila et al. (2022). The virulence hallmark amino acids (222A, 242I, 249Q, 253Q, S254, 256I, G258, T260, T269, A270, 272I, A278, 279D, 284A, 286 T, 294I, 299S, 318G, A321, 324L, and S330) were identified in our five vvIBDV strains. These hallmark amino acids were supposed to be responsible for the antigenicity and virulence of IBDV (Michel and Jackwood 2017; Lian et al. 2022; Akhila et al. 2022; Almaw et al. 2022). There were significant conserved amino acid substitutions at the surface P_BC_ loop (Y220F) in most Egyptian strains and P_DE_ loop (G254S) in all Egyptian vvIBDV strains. These amino acid substitutions were previously recorded to be stable in the Egyptian vvIBDV strains (Jackwood and Sommer-Wagner 2007; Michel and Jackwood 2017). The Egyptian vvIBDV strains have been found to exhibit a specific point mutation in loop PDE, where glycine is replaced by serine at position 254. This mutation is thought to be responsible for vaccination failure as previously documented by Michel and Jackwood (2017). Strain mans1 in our study was classified as a classical virulent IBDV, along with CEVAC® IBD L, Bursine® Plus, and VAXXITEK® HVT + IBD vaccines, as previously recorded by Yilmaz et al. (2019), who found that most of the studied cvIBDV were similar to vaccine strains. The majority of cvIBDVs, including vaccine strains, exhibited conserved amino acid substitutions, namely S217L, A222P, I242V, I256V, A270T, N293L, and S299N compared to vvIBDV strains. These results were in concurrence with Pikuła et al. (2021). These amino acid substitutions were also recorded in the new genogroup A9 (at IBDVs) except for the amino acid substitution S217L detected in most A1 cvIBDVs, not A9 atIBDVs. The genogroup A9 viruses have extra conserved amino acid substitutions (Q253H, D279N, A284T, and S330R) not detected in A1 or A3 viruses. The amino acids (217L, 222P, 242 V, 256 V, 270 T, 293L, 299N, 253H, 279N, 284 T and 330R) could be related to decrease virulence of IBDV strains as previously reported (Michel and Jackwood 2017; Lian et al. 2022; Akhila et al. 2022; Almaw et al. 2022). They reported that these amino acids were suggested to be responsible for the antigenicity and virulence of IBDV. Our cvIBDV strain (mans1) was hypothesized to be primarily related to the CEVAC® IBDL vaccine as they shared the same amino acid profile with 100% amino acid identity (0% diversity). Furthermore, the Bursine® Plus vaccine exhibited the highest dissimilarity to the mans1 strain, with a diversity of 7.59%. It contained distinct amino acid substitutions not found in other vaccines or strains within the same genogroup.

All strains identified in this study possess the vvIBDVs VP1 characteristic tripeptide TDN at the position 145-147aa. These results agree with Michel and Jackwood. (2017), Pikuła et al. (2020), and Akhila et al. (2022), which demonstrated the presence of a unique VP1 TDN tripeptide in vvIBDV. However, the non-vvIBD (genogroup B1) has amino acid substitutions D146E and N147G with varied amino acid substitutions T145N/S/D. These substitutions demonstrate that these strains were expected to originate from reassortment between vvIBDV and non-vvIBDV strains (Michel and Jackwood 2017). We also observed one additional conserved amino acid substitution, E246D, in non-vvIBD (genogroup B1), which was not previously recorded. In spite of ongoing vaccination efforts targeting IBDV, the virus persists in Egypt. Currently used vaccines appear ineffective against IBDVs within the major genogroups 1–3 (Michel and Jackwood 2017). There is a strong need for an updated classification and detailed analysis of the genogroups of IBDV, as well as its molecular characteristics. This is necessary in order to develop autogenous IBDV vaccines or virus-like-particles vaccines that can effectively combat the evolving nature of the virus (Michel and Jackwood 2017).

Interestingly, this study identified a novel strain that was classified as genotype A1B2 (cv-A/vv-B). Therefore, we compared the pathogenicity of reassortant (cv-A/vv-B) IBDV and vvIBDV strains identified in this study. The birds in G2 (cv-A/vv-B reassortant IBDV) and G3 (vvIBDV) developed typical IBD clinical signs and postmortem lesions, which were more severe in G3 than G2 as the degree of bursal destruction can vary significantly with the studied IBDV strain pathogenicity. These results were previously strengthened (OIE 2018). The mortality rate was 60% in cv-A/vv-B reassortant IBDV-inoculated chickens (G2) and 70% in vvIBDV-inoculated chickens (G3). The classical IBDV strains were previously recorded to be less virulent than the vvIBDV strains as the (classical) IBDVs caused 10–50% mortality, whereas vvIBDVs induce 50–100% mortality in experimentally infected chickens (OIE 2018; Dey et al. 2019). Wagari (2018) recorded 100% mortality in SPF chicks experimentally infected with vvIBDV, while Pikuła et al. (2021) observed 25 and 50% mortality in vvIBDV experimentally infected chicks, respectively. Tanimura (2022) recorded 40–60% mortality in vvIBDV-infected chickens and 0% mortality in cvIBDV-infected chickens. The cause of these IBDV pathogenicity and mortality differences may be due to the virulence of the strain, the dose of inoculated virus, and the breed of infected chickens (OIE 2018). In our study, the reason of five strains to be very virulent may be the different causes of vaccine failure, including the circulation of very virulent IBDV strains in Egypt, as the country is endemic with IBDV with minimal biosecurity measures (Mosad et al. 2020).

From previous data, we can conclude that the reassortment event increased the mortality and lesions caused by the cv-A/vv-B IBDV strain than that previously recorded for the classical strain. Segment-reassortment in IBDV may affect its pathogenicity as Wang et al. (2021) observed A2dB3 reassortment strain from two major epidemic strains (nVarIBDV and vvIBDV) with a characteristic nVarIBDV pathogenicity. However, Wang et al. (2022) identified a more virulent A2dB3 reassortment strain with 10% more mortality than that of the nVarIBDV. The typical histopathological lesions observed in the BF in chickens infected with vvIBDV, include follicular atrophy, varying degrees of lymphoid depletion, proliferation of fibrous tissue between follicles, and hemorrhage in the bursal tissue (Islam et al. 2008). The severity of lesions in the BF of IBDV-affected birds is associated with the degree of pathogenicity.

It is currently not feasible to determine the pathogenicity of the IBDV strain solely through histopathological examination, as the specific mechanisms by which different strains of IBDV cause distinct pathological changes are still not understood (Tanimura 2022). Bursal sections of birds infected with cv-A/vv-B reassortant IBDV showed vacuolation of the lymphoid follicles with moderate lymphocytic depletion. The results were previously identified (Tanimura 2022). As previously described, those infected with vvIBDV strains are characterized by an increase in acute inflammation and necrosis of lymphoid follicles (Ignjatovic 2004). Infection vvIBDV strains caused severe necrosis in the splenic follicles together with thymic medullary and cortical necrosis. Singh et al. (2015) showed that IHC staining is a precise, specific, rapid, and reliable method to demonstrate the IBDV antigen in the altered tissues due to IBDV infection, which concurs with gross or microscopic lesions. In the present study, the presence of viral antigen was also observed in the interfollicular connective tissue and the bursal epithelium. A similar reaction was also seen in the cortical lymphocytes of the thymus (Singh et al. 2015).

IBDV shedding in feces (5 and 7 days PI) was confirmed in G2 and G3 birds. These findings are consistent with Zhao et al. (2013), who reported that the IBDV-infected broilers began to shed IBDV in their feces 5- 12 days PI. Tanimura (2022) found that cvIBDV showed moderate BF lesions with no lesions in the thymus, lung, liver, kidney, and proventriculus of infected chickens. In this study, the bursal damage was more severe in vvIBDV-infected birds than in cvIBDV-infected birds, as confirmed by Tanimura (2022). On the 7th day PI, the antibody titer in G2 birds was higher than in G3 birds, potentially because G3 birds experienced more severe bursal damage and necrosis compared to G2 birds.

## Conclusion

This is the first report that classifies IBDV circulating in vaccinated broiler chicken farms according to the new classification scheme based on the analysis of both genome segments (hvVP2 and VP1) in Egypt. The comparative pathogenicity of reassortant IBDV and vvIBDV strains identified in this study developed typical IBD clinical signs, mortality, postmortem lesions, ELISA antibody titers, histopathology, immunohistochemistry, and lesion scores, which were more severe in vvIBDV than reassortant IBDV. It is highly recommended to continue molecular identification of circulating IBDV strains by sequencing both hvVP2 and either the partial VP1 gene or full-length sequences of the coding regions of both RNA segments in order to study genotypes and produce effective IBDV vaccines. However, serological studies of isolated genotypes and protection experiments are still required using representative strains of different genotypes.

## Electronic supplementary material

Below is the link to the electronic supplementary material.

Supplementary file1 (DOCX 24 KB)

## Data Availability

No datasets were generated or analysed during the current study.

## Abbreviations

BF: Bursa of Fabricius
IBDV: Infectious bursal disease virus
vvIBDV: Very virulent infectious bursal disease virus
cv: Classical virulent
hvVP2: VP2 hypervariable region
SPF-ECEs: Specific pathogen-free embryonated chicken eggs
IHC: Immunohistochemistry
RNA: Ribonucleic acid
RT-PCR: Reverse transcriptase polymerase chain reaction
EID50: 50% Embryo infective dose
PBS: Phosphate buffered saline
CAM: Chorioallantoic membrane
PI: Post-infection
PM: Post-mortem
RLQP: Reference laboratory for veterinary quality control on poultry production
RCF: Relative centrifugal force
