# Supplementary material for: Genotype classification and pathogenicity of infectious bursal disease virus circulating in vaccinated broiler chicken farms
Source: Vet Res Commun. 2024 Jul 30;48(5):3089–104. doi: 10.1007/s11259-024-10468-z (PMC11442545; doi:10.1007/s11259-024-10468-z)
Supplement: Supplementary file 1 — Supplementary file1 (DOCX 24 KB) [file 11259_2024_10468_MOESM1_ESM.docx]

**Table S1: Sample code, age of sampling, vaccine, age of vaccination, RT-PCR, sequenced samples, genotype, phenotype, virus titer, pathogenicity test, IBDV shedding, and ELISA of identified IBD viruses in this study.**

| **Sample code** | **Governorate** | **age of sampling**  **(days)** | **Vaccine** | **Vaccine strain** | **age of vaccination (day)** | **RT-PCR** | **sequenced samples** | **Genotype** | **Phenotype** | **Virus titer (EID_50_/ml )** | **Pathogenicity test**  **mortality, clinical signs and PM lesions** | **RT-PCR confirmation of experiment** | **IBDV shedding 5 and 7 days PI** | **ELISA (Antibody titer)**  **7 days PI** |
| --- | --- | --- | --- | --- | --- | --- | --- | --- | --- | --- | --- | --- | --- | --- |
| **mans1** | Dakahlia | 21 | VAXXITEK® HVT + IBD | Faragher 52 | 1 | Positive | √ | A1B2 | cv-A/vv-B reassortant IBDV | 6.5 log 10 | √ (60%)  IBDV typical signs and PM lesions | **Positive** | **Positive** | **6885**** |
| **mans2** | Dakahlia | 30 | Nobilis® Gumboro 228E | 228E | 12 | negative |  |  |  |  |  |  |  |  |
| **mans3** | Damietta | 19 | VAXXITEK® HVT + IBD | Faragher 52 | 1 | negative |  |  |  |  |  |  |  |  |
| **mans4** | Damietta | 25 | CEVAC® IBD L | Winterfield-2512 | 12 | Positive | √ | A3B2 | vvIBDV | 6 log 10 | √ (70%)  More severe IBDV signs and PM lesions | **Positive** | **Positive** | **6763** |
| **mans5** | Dakahlia | 18 | INNOVAX-ND-IBD® | *None | 1 | Positive | √ | A3B2 | vvIBDV |  |  |  |  |  |
| **mans6** | Gharbia | 21 | Nobilis® Gumboro 228E | 228E | 12 | negative |  |  |  |  |  |  |  |  |
| **mans7** | Dakahlia | 20 | VAXXITEK® HVT + IBD | Faragher 52 | 1 | Positive |  |  |  |  |  |  |  |  |
| **mans8** | Damietta | 30 | Bursine® Plus | Bursine 2 | 12 | Positive |  |  |  |  |  |  |  |  |
| **mans9** | Gharbia | 19 | VAXXITEK® HVT + IBD | Faragher 52 | 1 | Positive | √ | A3B2 | vvIBDV |  |  |  |  |  |
| **mans10** | Damietta | 27 | Nobilis® Gumboro D78 | D78 | 12 | Positive | √ | A3B2 | vvIBDV |  |  |  |  |  |
| **mans11** | Gharbia | 18 | INNOVAX-ND-IBD® | None | 1 | Positive |  |  |  |  |  |  |  |  |
| **mans12** | Dakahlia | 20 | CEVAC® IBD L | Winterfield-2512 | 12 | negative |  |  |  |  |  |  |  |  |
| **mans13** | Damietta | 29 | VAXXITEK® HVT + IBD | Faragher 52 | 1 | Positive |  |  |  |  |  |  |  |  |
| **mans14** | Gharbia | 28 | VAXXITEK® HVT + IBD | Faragher 52 | 1 | negative |  |  |  |  |  |  |  |  |
| **mans15** | Dakahlia | 19 | Bursine® Plus | Bursine 2 | 12 | Positive |  |  |  |  |  |  |  |  |
| **mans16** | Damietta | 22 | VAXXITEK® HVT + IBD | Faragher 52 | 1 | Positive |  |  |  |  |  |  |  |  |
| **mans17** | Gharbia | 24 | CEVAC® IBD L | Winterfield-2512 | 12 | Positive |  |  |  |  |  |  |  |  |
| **mans18** | Dakahlia | 28 | Nobilis® Gumboro 228E | 228E | 12 | negative |  |  |  |  |  |  |  |  |
| **mans19** | Damietta | 23 | INNOVAX-ND-IBD® | None | 1 | Positive |  |  |  |  |  |  |  |  |
| **mans20** | Gharbia | 25 | VAXXITEK® HVT + IBD | Faragher 52 | 1 | Positive |  |  |  |  |  |  |  |  |
| **mans21** | Gharbia | 26 | CEVAC® IBD L | Winterfield-2512 | 12 | Positive |  |  |  |  |  |  |  |  |
| **mans22** | Gharbia | 28 | VAXXITEK® HVT + IBD | Faragher 52 | 1 | Positive | √ | A3B2 | vvIBDV |  |  |  |  |  |
| **mans23** | Dakahlia | 22 | VAXXITEK® HVT + IBD | Faragher 52 | 1 | negative |  |  |  |  |  |  |  |  |
| **mans24** | Damietta | 19 | Bursine® Plus | Bursine 2 | 12 | Positive |  |  |  |  |  |  |  |  |
| **mans25** | Gharbia | 20 | VAXXITEK® HVT + IBD | Faragher 52 | 1 | negative |  |  |  |  |  |  |  |  |
| **mans26** | Dakahlia | 28 | Bursine® Plus | Bursine 2 | 12 | Positive |  |  |  |  |  |  |  |  |
| **mans27** | Damietta | 21 | CEVAC® IBD L | Winterfield-2512 | 12 | Positive |  |  |  |  |  |  |  |  |
| **mans28** | Gharbia | 23 | Nobilis® Gumboro 228E | 228E | 12 | Positive |  |  |  |  |  |  |  |  |
| **mans29** | Dakahlia | 27 | INNOVAX-ND-IBD® | None | 1 | negative |  |  |  |  |  |  |  |  |
| **mans30** | Damietta | 21 | INNOVAX-ND-IBD® | None | 1 | Positive |  |  |  |  |  |  |  |  |

*None: not mentioned in the product data

** The antibody titer was higher in G2 birds than G3 birds at the 7^th^ day PI *at P* value < 0.05.
